# Feasibility and Acceptability of Smartphone-Based Ecological Momentary Assessment of Alcohol Use Among African American Men Who Have Sex With Men in Baltimore

**DOI:** 10.2196/mhealth.4344

**Published:** 2015-06-17

**Authors:** Cui Yang, Beth Linas, Gregory Kirk, Robert Bollinger, Larry Chang, Geetanjali Chander, Daniel Siconolfi, Sharif Braxton, Abby Rudolph, Carl Latkin

**Affiliations:** ^1^ Johns Hopkins School of Public Health Department of Health, Behavior and Society Baltimore, MD United States; ^2^ Johns Hopkins School of Public Health Department of Epidemiology Baltimore, MD United States; ^3^ Johns Hopkins School of Medicine Baltimore, MD United States; ^4^ Pacific Institute for Research and Evaluation Beltsville, MD United States

**Keywords:** ecological momentary assessment (EMA), alcohol use, HIV, African American, men who have sex with men (MSM)

## Abstract

**Background:**

Alcohol use is a risk factor for the acquisition of human immunodeficiency virus (HIV) among African American men who have sex with men (MSM). Mobile phone-based ecological momentary assessments (EMA) could minimize bias due to retrospective recall and thus provide a better understanding of the social and structural context of alcohol use and its relationship with HIV-related risk behaviors in this population as well as other highly stigmatized populations.

**Objective:**

We describe the study design and the implementation, feasibility, reactivity, and acceptability of an EMA study of alcohol use and HIV-related behaviors among African American MSM in Baltimore.

**Methods:**

Participants were recruited through flyers and word-of-mouth in Baltimore from September 2013 to November 2014. Each participant was loaned an Android smartphone and instructed to respond to multiple prompts from the mobile app for 4 weeks. Data were collected through (1) random prompts delivered three times daily assessing participants’ location, activity, mood, and social context, (2) daily prompts capturing drinking and sex events occurring in the past 24 hours, and (3) event-contingent responses collecting participants’ self-reported episodes of drinking.

**Results:**

A total of 16 participants enrolled in the study. The current analyses focused on 15 participants who completed at least 24 days of follow-up (mean follow-up time 29 days; range 24-35 days). Study participants (N=15) were a median 38 years of age (range 27-62 years) with low levels of income and educational attainment. Ten individuals self-reported living with HIV/AIDS, over half reported drinking alcohol at least 2-3 times a week, and a third reported binge drinking (ie, 6 or more drinks on one occasion) on a weekly basis. Based on the Alcohol Use Disorders Identification Test (AUDIT) score, nearly half were classified as hazardous drinkers (score 8-15) and a fifth were likely dependent (score ≥16). A total of 140 participant-initiated events were reported, and 75% of 1308 random prompts and 81% of 436 daily prompts delivered were answered. Of seven devices used during the study, five were reported lost by participants. We did not observe strong reactivity effects, and self-reported acceptability to study procedures was uniformly favorable.

**Conclusions:**

This study provides evidence to support the feasibility and acceptability of using EMA methods for collecting data on alcohol use among African American men who have sex with men living in urban settings. These data provide the basis for future studies of EMA-informed mHealth interventions to promote the reduction of substance use and HIV risk-taking behaviors among African American MSM living in urban settings.

## Introduction

Epidemiological data suggest the highest rates of human immunodeficiency virus (HIV) infection in the United States are among African American men who have sex with men (MSM) [1]. The 2012 National HIV Behavioral Surveillance survey demonstrated that among HIV-infected MSM in Baltimore, 48% were African American and less than 20% were white. Alcohol use and its impact on HIV transmission and treatment are major public health burdens in many parts of the world. Reviews indicate that alcohol consumption is associated with HIV incidence [2]; furthermore, alcohol is a potential cause of poorer HIV outcomes [2] and is associated with lower adherence to HIV medications [3]. African American MSM report significantly more drinks per drinking day compared to MSM from other race/ethnicity groups [4]. Previous research in Baltimore found that, based on Alcohol Use Disorders Identification Test (AUDIT) scores, 22% of African American MSM were in the hazardous category (ie, AUDIT score 8-15) and 21% in the high risk/likely dependent category (AUDIT score ≥16) [5].

Most research on substance use (alcohol and illicit drugs) and HIV has relied on self-reported information over a period of time, which varies by study. A major limitation of this method of measurement is that it is affected by recall bias (reliability and accuracy), and it may not be context specific [6]. Information recall is affected by heuristics used in memory search and reconstruction, which can systematically bias participant responses [7,8]. Imprecise or inaccurate information can impede the advancement of knowledge regarding alcohol use and risky sex behaviors among highly stigmatized populations [9]. There is a need to improve the methodologies for behavioral data collection and to obtain a more detailed understanding of the relationship between alcohol use and HIV risk behaviors among key populations, especially African American MSM.

Mobile health (mHealth) opens new avenues for research on substance use and HIV, as ubiquitous technology allows for more frequent and close to real-time collection of behaviors, locations, and physiologies [10]. Ecological Momentary Assessment (EMA), an mHealth method that utilizes mobile technologies, such as a personal digital assistant (PDA) or mobile phone, allows participants to record their daily activities on the device in real time [11]. EMA minimizes biases, specifically recall bias, by requiring participants to immediately respond to random prompts or record specific events on a daily basis in their natural environments [12]. EMA is an especially suitable tool to study health behaviors, such as alcohol use, which are discrete and episodic behaviors, and is ideal for event-contingent recording [13]. Prior research suggests that MSM may use alcohol to cope with internal experiences (eg, stress associated with internalized homophobia) [14] and situational stimuli and cues (eg, social pressure to use), making EMA an excellent method to capture these fleeting states. Empirical data from previous research of EMA have shown a high compliance rate among diverse populations, including homeless persons with crack-cocaine addiction [15], heroin and cocaine users in treatment [16], ecstasy users who also engaged in use of alcohol, marijuana, cocaine and hallucinogens [17], and social drinkers [18]. Evidence has also shown that intoxicated participants could enter data accurately on a mobile device [13]. Finally, in studies assessing reactivity of substance-use recording in EMA, the possibility that the repeated assessments may affect the behavior under study and thus distort the findings, have not indicated strong reactivity effects [19,20].

While EMA methods have been used successfully, there have been few studies that employ these methods with African American MSM in everyday, community-dwelling, non-treatment settings. In response to this limitation, we developed EMA methods for near real-time characterization of alcohol use in individuals’ natural environments. In this paper, we characterize implementation barriers and examine the feasibility, acceptability, and reactivity of using intensive EMA methods among African American MSM living in urban settings.

## Methods

### Study Participants

Study participants were recruited through flyers and word-of-mouth in Baltimore, Maryland. Flyers were placed at the front desk of the research facility, where several other substance use and HIV projects were conducted. Inclusion criteria for this study were (1) at least 18 years of age, (2) self-reported African American race/ethnicity, (3) self-reported male sex, (4) self-reported having had sex with a male in the prior 30 days, (5) self-reported drinking alcohol at least once a week in the prior 30 days, (6) reported living within the Baltimore metropolitan area, and (7) able to understand and follow directions on how to use the mobile phone, as assessed by study staff in a one-on-one orientation session.

### Study Procedures

#### Recruitment/Enrollment

Between September 2013 and November 2014, we screened 25 individuals via phone or in-person by a trained study coordinator or principal investigator. Of those screened, 17 were eligible to participate in our study and 16 participants provided informed verbal consent to participate in the study. Participants were enrolled in 6 waves of data collection (2-3 participants/wave). Participants first completed a baseline interview at the research facility. They were then loaned a mobile phone (Samsung Galaxy S4), and the mobile phone was reused in each wave. Once a phone was lost, a replacement phone was acquired. To protect participants’ privacy, mobile phones were set back to factory setting and therefore all personal information was deleted. Before leaving the study office and beginning mobile data collection in the field, study participants were trained on how to use the device and app by the study coordinator and all participants were required to show their understanding of the mobile app by running a “demo” questionnaire.

#### Audio-Computer Assisted Standardized Interview Surveys

At baseline, all participants completed an audio computer-assisted self-interview (ACASI), which assessed sociodemographics (eg, age, education, employment, income, and homelessness), drug and sex behaviors (eg, self-reported alcohol, tobacco, and illicit drug use and number of sex partners), clinical diagnoses (eg, self-reported HIV status), and prior experience with mobile technology. Additional data collected at baseline included depressive symptoms assessed using the Center for Epidemiologic Studies Depression Scale (CES-D), and past week and hazardous drinking and likely alcohol dependence assessed via the AUDIT score (respective cut-offs: ≥8 and ≥16). Participants returned to the research center after 1 and 4 weeks of follow-up to complete additional ACASI surveys that collected information about their behaviors during the prior week (1-week assessment) or during the prior 30 days (4-week assessment). One of the goals of the ACASI surveys was to compare aggregated responses with real-time responses from the EMA data collection.

#### Mobile App

**Figure 1:**
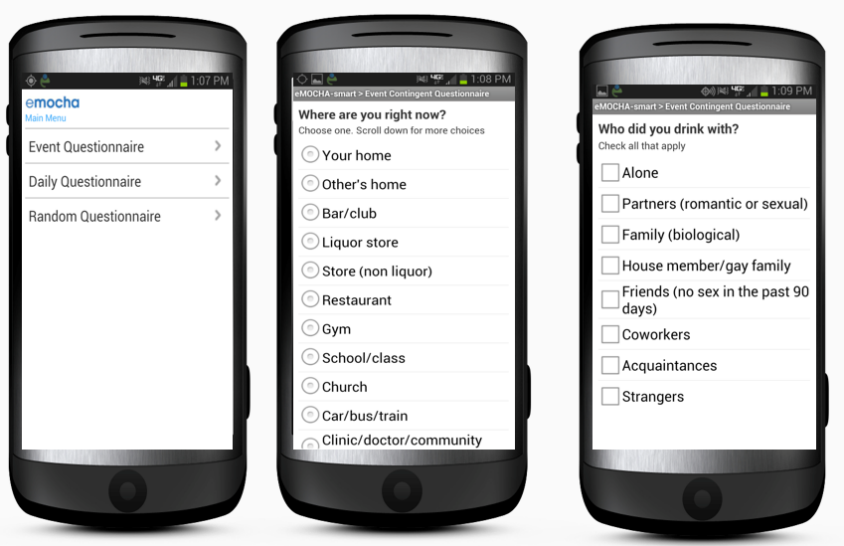
Screenshot of emocha event-contingent survey.

The mobile app used in this study, emocha, was created by the Center for Clinical Global Health Education at the Johns Hopkins School of Medicine. EMA surveys used in this study were adapted from prior research conducted by collaborators with drug using populations in Baltimore [21]. The following three types of EMA prompts were used in this study:

1. Random prompts: Three times daily, emocha sent an alert to the participant’s phone between 10 a.m. and 10 p.m. These random prompts asked about participants’ immediate mood, surroundings, and potential environmental cues that may trigger alcohol use. Participants answered no more than 46 questions in each of the random prompt surveys (including skip patterns).
2. Daily prompts: One daily alert at 9 a.m. was sent to participant’s phone. This survey consisted of questions that summarized activities during the previous 24 hours, including alcohol, other substance use, and sexual activities. The daily survey contained up to 98 questions, depending the number of alcohol drinks and number of sex partners reported during the past 24 hours.
3. Event-contingent entries: Participants were instructed to initiate an electronic entry every time they finished one episode of drinking, which was defined as a cluster of alcohol use in one sitting. Event-contingent surveys asked up to 40 questions concerning the location, alcohol use expectancy, drinking partner, types, and amount of alcohol, co-use of other substances, and current mood and stress level (see Figure 1).

Participants had 30 minutes after being prompted to complete surveys for both the random and daily prompts. After 30 minutes, the prompt was considered missed. Each day at 10 p.m., mocha uploaded the encrypted EMA data to a secure server and removed the data from the device.

### Qualitative Assessment

During the week 1 and week 4 visits, participants also completed 10-minute, semistructured interviews to provide feedback on their experience using the mobile phone and the emocha app. The qualitative interview guides had predefined main themes, but these guides were meant to be dynamic and allowed for new topics to emerge during the course of the interview. Important topics included (1) satisfaction and challenges with the study, (2) suggestions for future studies, and (3) ways the mobile phone may facilitate reducing alcohol use and promoting HIV risk reduction.

Participants received remuneration for attendance at study visits (Table 1), for providing adequate responses to weekly random and daily prompts, and for returning devices upon study completion. Participants were informed at enrollment that loss of two study devices would result in their dismissal from the study.

**Table 1:** Participant reimbursement/visit schedule (in USD).

	Intake	Week 1	Week 2	Week 3	Week 4	Total
Baseline visit	$10					
ACASI	$20	$20			$20	
EMA^a^		$25/$50	$25/$50	$25/$50	$25/$50	
Close out					$10	
Smartphone return^b^					$50/$100	
Total	$30	$45-$70	$25-$50	$25-$50	$105-$180	$230-$380

^a^Participants were paid US $50 every week for answering 80% of their alarms or US $25 every week for answering 60% of their alarms. They received no bonus for answering less than 60% of their alarms or if their phone was uncharged.

^b^Participants received US $100 at close out for returning their original phone or US $50 for returning their replacement. They would be excused from the proposed study if they lost their replacement phone.

The Institutional Review Board at Johns Hopkins University Bloomberg School of Public Health approved the study protocol and a Certificate of Confidentiality was obtained through the National Institute of Allergy and Infectious Diseases.

### Data Analysis

Descriptive statistics were used to examine characteristics of participants and study compliance (eg, days of follow-up, random and daily prompt response rates, EMA survey completion time, and device loss rate). Feasibility was assessed through participant retention, days of follow-up, device loss rate, response rates to EMA surveys, and amount of time needed to complete each EMA survey. We also assessed the number of questions completed in each survey.

Reactivity analysis was conducted by examining any correlation between day of study and total number of drinks reported in daily surveys. Repeated measures were accounted for in linear regression models using Generalized Estimating Equations [22]. We summed the number of drinks each individual reported consuming per day in the daily prompts and plotted this against the day of study. We used a non-parametric lowess curve, which is able to show a relationship between variables and any trends that may exist in the data. All quantitative analyses were performed using Stata version 13.0.

For the qualitative evaluation, we identified core consistencies and meanings in the data through careful repeated reading of interview texts. We labeled sections of text based on themes and particular domains of interest related to feasibility and acceptance. Results were summarized by main themes and reviewed by investigators continually throughout the study, with the goal of identifying strategies to refine the emocha design in preparation for a subsequent data collection.

## Results

### Baseline Characteristics of Participants

Of the 16 participants enrolled, one participant was lost to follow-up 1 day after the baseline visit. The current analyses focused on 15 participants who completed at least 24 days of follow-up (median 29, interquartile range 27-31). Of these 15 participants, 5 saw the study flyers and 10 heard about our study from other people. The baseline characteristics of 15 participants were summarized in the Table 2.

**Table 2:** Baseline characteristics of participants (N=15).

Characteristics		n (%)
Age, median (IQR)		32 (29-45)
At least grade 12 or GED education		13 (87)
Full/part time job		2 (13)
<US $10,000 income (last year)		8 (53)
Homeless (past 6 months)		4 (27)
Arrested (past 6 months)		2 (13)
HIV positive (self-report)		10 (67)
CES-D score, median (IQR)		32 (21-44)
Depressive symptoms (CES-D>20)		12 (80)
**Frequency of cigarette use (past 30 days)**		
	Never	3 (20)
	Once a week	1 (7)
	A few times a week	2 (13)
	Every day	9 (60)
Have smoked crack/cocaine/heroin/inject drugs to get high (past 3 months)		4 (27)
Often or always smoke marijuana while drinking alcohol		7 (47)
**Frequency of alcohol use**		
	Monthly or less	1 (6)
	2-4 times a month	6 (40)
	2-3 times a week	4 (27)
	4 or more times a week	4 (27)
Frequency of binge drinking at least weekly		5 (33)
AUDIT score, median (IQR)		9 (6-14)
Hazardous drinker (AUDIT score: 8-15)		7 (47)
Probable alcohol dependence (AUDIT score≥16)		2 (20)
Number of sex partners (past 30 days), median (IQR)		2 (1-5)
Have owned a mobile phone (past 6 months)		14 (93)
Currently using a smartphone		11 (79)

### Feasibility Assessment

Overall, 15 participants provided 436 days of observation (mean 29 days; Table 3). Seven participants enrolled in the study over 4 weeks (ie, 28 days) due to scheduling. Of the 15 participants, 2 completed 4 weeks of EMA entries but failed to return to the clinic for the 4-week visit and to return the phone. Two more participants were unable to complete week 4 of EMA surveys due to phone loss (n=1) and incarceration (n=1); however, both men completed the last clinic visit. A total of seven phones were issued to participants, and phones were re-used for multiple waves of data collection. At the end of the study, five phones were either reported lost by participants (n=2) or were unable to be retrieved by study staff due to loss to follow up with participants (n=3). One participant reported losing his phone on the last day of follow-up. The last phone was lost during the fourth week of EMA data collection, but the participant did not report the phone as lost until the last day of the study.

**Table 3:** Days of follow-up and device loss (participants were enrolled in 6 waves of data collection [2-3 participants/wave]. Phone was reused in each wave. Once a phone was lost, a replacement phone was acquired).

	Baseline	Week 1	Week 2	Week 3	Week 4		Days	Device
Participant 1	X	X	X	X	X		29	lost
Participant 2	X	X	X	X	X	X	30	returned
Participant 3	X	X	X	X	X	X	29	returned
Participant 4	X	X	X	X	X	X	27	returned
Participant 5	X	X	X	X	X	X	31	returned
Participant 6	X	X	X	X	X		28	lost
Participant 7	X	X	X	X	X	X	27	returned
Participant 8	X	X	X	X	X	X	31	returned
Participant 9^a^	X						1	lost
Participant 10	X	X	X	X	X	X	27	returned
Participant 11	X	X	X	X	X	X	35	returned
Participant 12	X	X	X	X	X	X	32	returned
Participant 13	X	X	X	X		X	24	lost
Participant 14	X	X	X	X		X	24	returned
Participant 15	X	X	X	X	X	X	35	returned
Participant 16	X	X	X	X	X	X	27	lost

^a^Excluded from the current analyses.

Response rates, time to complete EMA survey and number of questions completed over the 4-week study period are summarized in Table 4. A total of 436 daily prompts, which were initiated at 9 a.m. every day, were sent to participant’s mobile phones. In all, 352 daily prompts were completed resulting in an overall compliance rate of 80.7%. The compliance rate of daily survey completion ranged from 62.5% to 100% among all participants. Table 4 describes the peak compliance rate in week 3 (92.4%) followed by a drop in week 4 (63.6%).

A total of 1308 random prompts were sent to participants’ mobile phones over follow-up and 968 were completed. This represents an overall compliance rate of 74%, translating to an average of 2.22 random-prompt responses per day per person. Table 4 shows the compliance rate per week as steady in the first 3 weeks, followed by a drop-off in week 4. Among all participants, the compliance rate of random prompts ranged from 48.1% to 98.9%.

The 15 participants of the current study reported a total of 140 drinking events over follow-up through emocha. The average number of self-reported drinking events per person was 9, ranging from of 2 to 32 reports over 4 weeks of follow-up. Of note, 40% of drinking events were reported in week 1 and the number reported per week decreased at each week over the course of the study.

We assessed completion time as the number of minutes elapsed from initiation of each survey to synchronization with the server. The average time to finish the daily survey was 1.43 minutes, the average time to complete the random survey was 1.15 minutes, and the average time needed to complete the event survey was 1.52 minutes. In both daily and random surveys, we observed a trend of a learning curve so that initially participants took longer to complete the survey. In week 4, it took the participants an average of 1 minute to finish each survey. Taken together, the amount of time it took to complete one daily survey and three random surveys averaged 4.88 minutes per day plus any additional time to fill out event surveys if drinking occurred. We also assessed the number of questions participants completed in different the types of surveys per week over 4 weeks. There were no significant changes over time in terms of number of completed questions in both random and event surveys. Although the change was statistically significant (*P*=.006) in the daily survey, the magnitude of change was from 19.33 in week 1, 15.10 in week 2, 15.21 in week 3, and 15.91 in week 4.

**Table 4:** Response rates, time to complete EMA survey, and number of questions completed.

	Average response rate			Time to finish EMA survey in minutes, median (IQR)			Number of questions completed in each survey, mean (SD)		
	Daily survey	Random survey	Event, n	Daily survey	Random survey^a^	Event survey	Daily survey	Random survey	Event survey
Overall	80.7%	74%	140	1.43 (0.91-2.53)	1.15 (0.83-1.60)	1.52 (1.15-2.10)	16.36 (9.24)	18.06 (3.15)	18.92 (1.04)
Week 1	85.7%	81.6%	58	2.64 (1.57-3.68)	1.48 (1.11-1.99)	1.97 (1.55-2.78)	19.33 (12.01)	17.96 (3.14)	19.14 (1.32)
Week 1	83.8%	84.8%	28	1.28 (0.89-2.19)	1.17 (0.83-1.71)	1.18 (1.06-1.58)	15.10 (8.98)	18.24 (3.37)	18.75 (0.79)
Week 3	92.4%	80.9%	32	1.19 (0.84-1.98)	1.06 (0.79-1.35)	1.29 (1.11-1.90)	15.21 (7.03)	17.75 (2.82)	18.75 (0.62)
Week 4	63.6%	52.1%	22	1.05 (0.81-1.84)	0.95 (0.70-1.24)	1.16 (0.89-1.55)	15.91 (7.73)	18.33 (3.24)	18.82 (0.91)
*P* value				<.001	.28	<.001	.006	.18	.23

^a^Data only available for ID8-ID16.

### Reactivity Assessment

**Figure 2:**
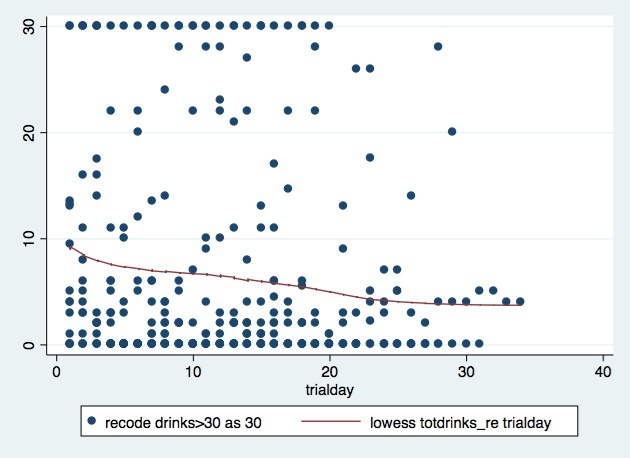
Trend in reporting number of drinks in daily survey.

The median number of drinks per day per person was 2 (IQR 0-6). Cases with number of drinks per day over 30, which represented 9% of all cases, were recoded as 30 for further analysis. The correlation between number of drinks per day and days of study was -.015 (*P*=.01). We plotted the number of drinks per day against the day of study, and each dot represents each individual’s self-reported number of drinks. As shown in Figure 2, there was a trend towards decreasing alcohol use over the course of the study, and it flattened out after 25 days.

### Acceptability Assessment

As seen in Table 5, most participants reported that the phones were “easy or very easy” to use and that the reporting burden was “just right or not enough”. Comprehension of survey questions was also high (85%-94% reported “most” or “all made sense”). The majority (87%-93%) reported being mostly or extremely confident that their privacy would be protected. Finally, 20%-31% of participants indicated that answering questions about drinking made them want to drink less.

**Table 5:** Acceptability survey.

		Week 1 (n=15), n (%)	Week 4 (n=13), n (%)
**In general, how easy is it to use the smartphone?**			
	Very easy	13 (87)	13 (100)
	Easy	2 (13)	0
	Difficult	0	0
	Very difficult	0	0
**What do you think about the number of times that your alarm goes off every day?**			
	Not enough	1 (6)	1 (8)
	Just right	14 (94)	12 (92)
	A little too much	0	0
	Too much	0	0
**Do the questions on the phone make sense to you?**			
	None of the questions make sense to me	0	0
	Some of the questions do not make sense to me	1 (6)	2 (15)
	Yes, most of them make sense to me	4 (27)	5 (39)
	Yes, all of them make sense to me	10 (67)	6 (46)
**Do you feel comfortable carrying the smartphone?**			
	Extremely comfortable	14 (93)	10 (77)
	Mostly comfortable	0	2 (15)
	Somewhat comfortable	1 (7)	1 (8)
	Not too comfortable	0	0
	Not comfortable at all	0	0
**Do you feel confident that the information collected will only be seen by researchers and not used against you?**			
	Extremely confident	12 (80)	11 (86)
	Mostly confident	1 (7)	1 (7)
	Somewhat confident	2 (13)	1 (7)
	Not too confident	0	0
	Not confident at all	0	0
**Do you feel that the size of the device is:**			
	Too small	1 (7)	2 (15)
	A good size	14 (93)	11 (85)
	Too big	0	0
**Does answering questions about drinking make you want to drink more, less, or about the same?**			
	More	1 (7)	1 (7)
	Less	3 (20)	4 (31)
	The same	11 (73)	8 (62)

### Qualitative Assessment

In qualitative interviews, participants provided positive feedback concerning the study methods. Familiarity with the technology seemed to have helped participants navigate the study as participants stated “I have the same phone, so I like it” (Participant 8) and “easy to use, similar to my own phone” (Participant 13). Participants enjoyed the technology as part of the research, as one participant stated “fun to answer the questions on phone, much easier” (Participant 5). Participants felt the mobile technology may have even increased their engagement in the study, as participants stated “fun study which makes people more engaged and willing to participate” (Participant 13) and “good way to answer questions without coming to a clinic, it is cool” (Participant 14). One participant mentioned “The questions also make you more cognizant of your surroundings and habits (anxiety level, seeing things happen, amount of drinking)” (Participant 13).

Participants also expressed concerns and provided suggestions for the study procedures and future studies. For participants reporting that they already owned a smartphone, the study phone may have been a burden. As one participant stated, “two phones are too much (to carry)” (Participant 8). Another participant suggested that in future studies, study staff should “install apps (emocha) on my phone” (Participant 15). Participants also reported, “the battery life dies fast” (Participant 14), “volume is too low to hear the alarms and snooze time should be longer” (Participant 7), “[the apps] need to be more personalized” (Participant 13).

Participants expressed their interest in keeping the study phone as they stated “it [study phone] can’t be kept” (Participant 12) and “had to give it [study phone] back” (Participant 15). Participants also suggested extending the hours of data collection to better capture times when people are more likely to be out drinking. For example, one participant suggested that we “increase the time on weekends to around 1 or 2 [am]. That’s when the clubs close and if you do that, you would get some good data” (Participant 13), and another suggested that it “May be a good idea to have the alarm period extended to 11-12, people may be getting ready for parties” (Participant 14). In addition to data collection, participants felt the “smartphone can be a good tool to deliver health messages” (Participant 5).

## Discussion

### Principal Findings

To our knowledge, this is the first study to evaluate the use of a mobile app–based EMA to prospectively capture alcohol use among African American men who have sex with men living in urban settings. Despite challenges, this study provides evidence to support the feasibility and acceptability of using EMA methods for collecting data on alcohol use in this population.

Given the highly demanding protocol in EMA, we were particularly sensitive to participation burden. Our study protocol included 3 random prompts and 1 daily prompt per day, which represents a lower or moderate participant burden as compared to previous EMA studies in substance using populations [16,21]. In both daily and random surveys, we observed a trend of a learning curve, in that participants initially took longer to complete surveys. Overall, our data demonstrate that participants spent on average less than 5 minutes per day to compete mandatory EMA surveys. Event-driven surveys required an additional 1-2 minutes per drinking episode reported. The quality of EMA data depends heavily on participants’ compliance to prompts and timeliness of recording episodes of the desired event (ie, alcohol use). Noncompliance leads not only to missing data but can even introduce bias in the data collected [12]. In the current study, participants answered 74% of random prompts and 80.7% of daily prompts, which is comparable to response rates reported in previous EMA studies (50%-90%) [16,23,24]. This finding is consistent with what participants reported in the acceptability survey, as they were very clear in reporting that study procedures were not overly burdensome. Overall, findings from the current study represent a moderate burden to participants enrolled in the study.

Assessing compliance through the recorded events (eg, alcohol use) is much more challenging. There is often no way to independently assess or verify whether participants failed to report the events that have actually occurred. The idea of providing incentives for reporting substance use events is debatable and needs further evaluation. In the current study, 40% of drinking events were reported in week 1. Although the reactivity analysis found a significant decrease in reporting of number of drinks per day, the magnitude of decrease was minor (-.015). Taken together, underreporting of alcohol events through event-contingent surveys is expected in the current study. More research should explore good participant management procedures that can yield high compliance [13], such as a regular reminder to participants to report their drinking events when they have an onsite visit or through text messages to their phones. Future studies could consider using biochemical markers of alcohol, such as transdermal alcohol sensors as a way to objectively validate self-reported alcohol use (and compliance).

One of the challenges associated with EMA is exhaustion of participants within the study period due to the highly demanding research protocols, which can diminish the level of participation. In our analyses of weekly response rates, we did find some evidence of exhaustion, as the response rates to both daily and random prompts dropped significantly in week 4. These results may signify that further examination of the assessment windows, such as shorter follow-up are necessary. Additionally, we had significant loss to follow-up, which mostly occurred in week 4. In the future, studies should provide better monitoring to seek a better understanding of exhaustion with similar populations. We were able to find out that one participant was not be able to complete EMA in week 4 due to a brief incarceration. African American MSM living in urban settings may experience unique social and structural challenges, such unemployment, low-income status, incarceration, and community violence. These sociostructural factors may operate independently or together in a dynamic fashion to create a context in which they experience challenges or inabilities to engage in prevention and treatment programs [25]. Impacts of future mHealth research and programs will come from a better understanding of broader social contexts where mHealth is implemented.

Another challenge is related to an issue that concerns all EMA studies of substance use, namely the hours of coverage for EMA assessments [13]. In the current study, EMA assessments occurred between 10 a.m. to 10 p.m., in order to avoid alarming participants when they are asleep. However, as some participants suggested, these times of day may not be representative of hours in which drinking occurs, particularly as alcohol consumption tends to occur later in the night, and mood, activity, and social settings vary by time (eg, weekdays vs weekends). Thus, it is important for future studies to assess the full range of an individual participant’s waking hours [26] and to possibly provide personalized hours of coverage of EMA for each participant. In Epstein et al’s research with cocaine- and heroin-abusing outpatients who were being treated with methadone, typical waking hours were programmed on a weekly basis when participants were issued the device [16]. With technology development, personalized EMA could be executed remotely.

Device loss posed a major challenge in our study, as five of seven devices issued to participants were lost. Participants were informed at enrollment that they would be dismissed from the study after losing two devices. There is a concern that when using mobile devices among impoverished populations, participants might sell the devices [21]. Therefore, we provided an incentive (US $100) for returning the devices upon completion of the study. However, we later realized that the street value for the Samsung Galaxy 4 can be more than the incentive. Several participants expressed their disappointment at returning the study phones in qualitative interviews. Future studies may consider the option of installing the mobile app on participants’ own phones or letting participants keep the study phone instead of monetary incentive or using a less well-known brand of mobile phone. Additionally, future research could utilize the remote inactivation feature if mobile phones are stolen or misplaced.

### Limitations

This is a preliminary feasibility study, and so several limitations need to be addressed in future research. Given the small sample size, the current study does not have enough statistical power to detect the significant differences in sociodemographic, behavior, or clinical characteristics between participants with higher response rates and those with lower response rates. Research involving larger samples is needed to explore various factors associated with variation in compliance rates that can be used for targeted EMA training to enhance compliance. Despite the less restrictive inclusion criteria of our study, we were able to enroll participants with varied alcohol use, including 7 hazardous drinkers and 3 likely alcohol dependent. Our limited sample size, however, may not extend to problematic alcohol users with more intense alcohol use patterns. Our study confirmed previous findings that did not demonstrate strong reactivity from EMA assessment of substance use [20]; however, future research should generate more rigorous evidence.

### Conclusions

In conclusion, findings from our study demonstrate that EMA methods are feasible and acceptable approaches for data collection among African American men who have sex with men. Eliminating health disparities and reversing HIV epidemic trends will require innovative combination prevention approaches to reduce high-risk behaviors, including substance use, expanded HIV testing, and increased linkage to and retention in care. The high ownership of mobile phones among minority MSM may provide a promising platform for data collection and the delivery of substance use and HIV risk reduction messages to this hard-to-reach population [27]. Using EMA data can identify individualized sets of triggers to be used to further tailor “ecological momentary intervention (EMI)” content and delivery [28]. These methods could reinforce the systematic use of prevention or treatment components in real-world settings.

## Abbreviations

ACASI: audio computer-assisted self-interview
EMA: ecologic momentary assessment
emocha: electronic Mobile Comprehensive Health App
HIV: human immunodeficiency virus
MSM: men who have sex with men
